# Personal Recovery in the General Population: Comparison of Psychometric Properties of the Brief INSPIRE-O in Those With and Without Common Mental Disorders

**DOI:** 10.1177/10731911251367122

**Published:** 2025-09-16

**Authors:** Xingjian Ruan, Femke Vergeer-Hagoort, Margreet ten Have, Annemarie I. Luik, Marlous Tuithof, Ernst Bohlmeijer, Peter M. ten Klooster

**Affiliations:** 1 University of Twente, Enschede, The Netherlands; 2 Netherlands Institute of Mental Health and Addiction (Trimbos Institute), Utrecht, The Netherlands; 3 Erasmus MC University Medical Centre, Rotterdam, The Netherlands

**Keywords:** personal recovery, measurement invariance, psychological well-being, common mental disorders, psychometric validation

## Abstract

The 5-item Brief INSPIRE-O instrument, based on the Connectedness, Hope, Identity, Meaning in Life, and Empowerment framework, is a novel tool to assess personal recovery. Although initially developed for clinical populations, its conceptual alignment with core dimensions of psychological well-being suggests its potential applicability to a broader audience. The current study aimed to examine its validity, reliability, and measurement invariance across people with and without common mental disorders (CMDs). The scale was administered in a Dutch general population sample (*n* = 5,451). Confirmatory factor analyses supported a unidimensional structure with robust factor loadings and scalar invariance across individuals with and without CMDs in the past year. In addition, the Brief INPSIRE-O showed acceptable reliability (ω = .71–.78) and the expected pattern of correlations with other health indicators supported its construct validity. In conclusion, the Brief INSPIRE-O appears to be a psychometrically sound measure of positive psychological functioning that can be validly used and compared across people with and without CMDs.

## Introduction

In the recovery of mental illness, two main categories of recovery have been proposed: clinical recovery and personal recovery (Slade et al., 2008). While clinical recovery emphasizes symptom remission and functioning restoration, personal recovery focuses on the development of a meaningful life despite ongoing mental health challenges (Slade et al., 2008). Personal recovery is a deeply personal, unique process of changing one’s attitudes, values, feelings, goals, skills, and/or roles. It is a way of living a satisfying, hopeful, and contributing life even with limitations caused by illness (Anthony, 1993). Contemporary mental health services increasingly recognize the importance of integrating both clinical and personal recovery approaches, as research indicates that symptom improvement alone may be insufficient for overall well-being and life satisfaction (Oades, 2012; Slade, 2009).

Following several conceptual frameworks of personal recovery (e.g., Lapsley et al., 2002; Slade, 2009), an overarching framework was proposed by Leamy et al. (2011), based on a systematic review and narrative synthesis of descriptions and models of personal recovery. They suggested personal recovery comprises five key processes: Connectedness (e.g., relationships and support from others), Hope and optimism about the future (e.g., belief in the possibility of improvement), Identity (e.g., rebuilding a positive sense of identity), Meaning in life (e.g., meaningful life and social roles), and Empowerment (e.g., personal responsibility). This Connectedness, Hope, Identity, Meaning in Life, and Empowerment (CHIME) model is a comprehensive model of personal recovery and has been used to review the concept validity of existing personal recovery measures (Leamy et al., 2023; Shanks et al., 2013).

Several instruments of personal recovery have been developed and tested. A systematic review of existing measures of personal recovery identified thirteen instruments (Shanks et al., 2013). Only the items of the Questionnaire About the Process of Recovery (QPR), which was developed aiming to measure recovery in people with experience of psychosis (Neil et al., 2009), were completely mapped to the CHIME categories, but with uneven coverage. Williams et al. (2015) were the first to develop a measure of staff support for personal recovery fully based on the CHIME framework, named INSPIRE. The Support section of the INSPIRE contains 20 items, with four items covering each of the five CHIME domains of personal recovery. In addition, a 5-item Brief INSPIRE was developed with one item chosen from each CHIME domain based on item performance. Both the full and Brief INSPIRE showed adequate factorial validity, convergent validity, internal consistency, test–retest reliability, sensitivity to change, and feasibility (Schön et al., 2015; Williams et al., 2015). The INSPIRE was also evaluated across different countries, such as Sweden (Schön et al., 2015) and Japan (Kotake et al., 2020). Although some studies did not statistically support the existence of five CHIME dimensions of the Full INSPIRE (Kotake et al., 2020; Šaltytė Benth et al., 2023), the Brief INSPIRE with a one-factor solution would not be impacted by this critique (Williams et al., 2015).

Recently, a modified Brief INSPIRE was developed, coined the Brief INSPIRE-O (Moeller et al., 2023). While the original INSPIRE instruments assess recovery support from a practitioner, the Brief INSPIRE-O was specifically designed to assess the actual self-perceived level of personal recovery outcomes. For this purpose, the Brief INSPIRE was modified by removing the reference to the practitioner in each item. For example, the item “My worker helps me to feel good about myself” was changed into “I feel good about myself.” The Brief INSPIRE-O has shown acceptable scalability as a unidimensional measure and strong psychometric properties in patients receiving treatment for diverse mental health problems (Moeller et al., 2024). However, the items of the Brief INSPIRE-O are potentially not only applicable to people with mental illness, as they refer to positive psychological functioning in general, irrespective of mental illness. Conceptually, the CHIME framework of personal recovery overlaps highly with the framework of psychological well-being used in positive psychological approaches (Resnick & Rosenheck, 2006; Slade, 2010). Psychological well-being encompasses autonomy, environmental mastery, personal growth, positive relations with others, purpose in life, and self-acceptance (Ryff, 1989; Ryff & Keyes, 1995). Specifically, among these dimensions, autonomy and environmental mastery overlap with the Empowerment domain of CHIME, personal growth with Hope, positive relations with others with Connectedness, purpose in life with Meaning, and self-acceptance with Identity. Kraiss et al. (2019) indeed found that personal recovery in people with bipolar disorder, as measured by the QPR, was highly correlated with a well-established instrument measuring psychological well-being (*r* = .80). Van Eck et al. (2023) additionally found that personal recovery as measured by the Recovery Assessment Scale in nonaffective psychosis, unaffected siblings, and healthy controls had similar associated factors such as positive self-image, schemas, and functional coping strategies.

The first study describing the Brief INSPIRE-O already used the measure in a large Danish general population sample (Moeller et al., 2023), suggesting that its items measure a general concept of psychological well-being that is relevant for all people. Recently, Swildens et al. (2025) validated the Dutch version of the Brief INSPIRE-O specifically for individuals with severe mental illness, demonstrating good psychometric properties in this clinical population. However, no studies to date have thoroughly examined whether the scale can indeed be used as a generic and valid measure of psychological well-being among people with and without mental illness experiences. Therefore, this study aimed to further validate and compare the Brief INSPIRE-O in people with and without common mental disorder (CMD) in a large Dutch adult sample (Ten Have, Tuithof, Van Dorsselaer, Schouten, & De Graaf, 2023). First, the unidimensional structure of its items was more thoroughly tested using confirmatory factor analysis (CFA). Next, its measurement invariance across subgroups of people with and without a lifetime or past 12-month CMD was tested using multigroup CFAs. Measurement invariance indicates whether a questionnaire measures the same construct in the same way across different groups and is a prerequisite for comparing questionnaire scores across groups. Finally, we further assessed and compared the reliability and construct validity of the scale across people with and without CMD.

To examine the construct validity of the Brief INSPIRE-O, associations with work loss, burnout, life satisfaction, loneliness, and health-related quality of life were assessed. Based on previous research and theoretical considerations, we hypothesized specific patterns of associations between Brief INSPIRE-O scores as an indicator of psychological well-being and the included measures in the total sample. For work loss, we expected a small negative correlation, as previous studies have found weak associations between psychological well-being and sick leave (Soane et al., 2013; Straume & Vittersø, 2015). Regarding burnout symptoms, we hypothesized a medium negative correlation, based on previous findings about the relationship between psychological well-being and burnout (Beaumont et al., 2016; Rehman et al., 2020; Wang et al., 2017). For loneliness, we anticipated a large negative correlation, based on a meta-analysis demonstrating strong associations between loneliness and psychological well-being (Park et al., 2020). For life satisfaction, we expected a large positive correlation supported by meta-analytic evidence showing similar magnitudes of association between meaning in life (a key point of psychological well-being) and life satisfaction (Martinez-Calderon et al., 2023). For the Medical Outcomes Study Short Form-36 subscales, we hypothesized a gradient of associations, with a large positive correlation for mental health and medium positive correlations for social functioning and role-emotional functioning compared to weak positive correlations with physical health-related subscales (bodily pain, physical functioning, and role-physical). This expected pattern was based on meta-analytic evidence showing differential associations between psychological well-being and mental/physical health (Czekierda et al., 2017; Martinez-Calderon et al., 2023).

## Methods

### Sample and Procedure

Data were drawn from the Netherlands Mental Health Survey and Incidence Study-3 (NEMESIS-3), a large-scale psychiatric epidemiological cohort study of individuals aged 18 to 75 years sampled from the Dutch general population (Ten Have, Tuithof, Van Dorsselaer, Schouten, & De Graaf, 2023). The study employed a multistage, stratified random sampling design in which 148 of the 355 Dutch municipalities were systematically selected, stratified by four geographic regions and five levels of population density. Professional interviewers conducted structured face-to-face interviews in participants’ homes using laptop computer-assisted personal interviewing technology. Data was collected between November 2019 and March 2022, with an average interview duration of 91 min. The recruitment process achieved an overall response rate of 54.6%, resulting in 6,194 completed interviews. The current analysis included 5,451 participants who permitted their data to be shared with and analyzed by third parties. Full details of the NEMESIS-3 study design, sampling procedures, and sample characteristics can be found in Ten Have, Tuithof, Van Dorsselaer, Schouten, and De Graaf (2023).

### Measures

#### Diagnostic Assessment

The presence of lifetime and past 12-month mental disorders was assessed using a slightly modified version of the Composite International Diagnostic Interview (CIDI) version 3.0 (Ten Have, Tuithof, Van Dorsselaer, Schouten, & De Graaf, 2023). The CIDI 3.0 is a structured lay-administered diagnostic interview developed for the WHO World Mental Health Survey Initiative, with demonstrated good validity compared to blinded clinical reappraisal interviews using the Structured Clinical Interview for DSM-IV (Haro et al., 2006). The instrument was adapted to enable the assessment of DSM-5 disorders while maintaining comparability with DSM-IV diagnoses from the previous NEMESIS study. Details on the specific adaptations made to the CIDI 3.0 to accommodate DSM-5 criteria can be found elsewhere (Ten Have, Tuithof, Van Dorsselaer, Schouten, Luik, et al., 2023).

In NEMESIS-3, the following CMDs were assessed: mood disorders (major depressive disorder, persistent depressive disorder, and bipolar disorder), anxiety disorders (social phobia, specific phobia, panic disorder, generalized anxiety disorder, and agoraphobia), substance use disorders (alcohol and drug use disorders), and attention-deficit/hyperactivity disorder (ADHD). For the current analyses, two primary diagnostic variables were computed based on the CIDI diagnoses: lifetime disorder (0 = *no disorder*, 1 = *any lifetime mood, anxiety, substance use disorder*, or ADHD) and past 12-month disorder (0 = *no disorder*, 1 = *any mood, anxiety, substance use disorder*, or ADHD in the past 12 months). For clarity in subsequent analyses, we refer to the past 12-month disorders as current disorders, reflecting their recent manifestation within the year preceding assessment.

#### Brief INSPIRE-O

The Brief INSPIRE-O is a 5-item measure assessing personal recovery outcome, modified from the Brief INSPIRE support measure (Williams et al., 2015). Each item represents one domain from the CHIME framework of personal recovery (Leamy et al., 2011): Connectedness (“I feel supported by other people”), Hope (“I have hopes and dreams for the future”), Identity (“I feel good about myself”), Meaning and purpose (“I do things that mean something to me”), and Empowerment (“I feel in control of my life”). Items are rated on a 5-point Likert scale from 0 (*not at all*) to 4 (*very much*). Following scoring guidelines (Moeller et al., 2023), raw scores were summed and multiplied by 5 to give a total score ranging from 0 to 100, with higher scores indicating better personal functioning. Internal consistency in the current sample *was* acceptable (α = .75).

#### WHO Disability Assessment Schedule

The WHO Disability Assessment Schedule (Von Korff et al., 2008) consists of 3 items measuring the number of days in the past 30 days where respondents: (a) were totally unable to work or carry out normal activities, (b) had to cut back on work or normal activities, and (c) had reduced quality or attention to detail in their work or normal activities. The total work loss score was calculated by counting days of total inability to work at full weight, while days with reduced capacity (items 2 and 3) were weighted at 0.5, following the methodology of Von Korff et al. (2008). The total score ranges from 0 to 30 days per month.

#### Utrecht Burnout Scale

The Utrecht Burnout Scale emotional exhaustion subscale (Schaufeli et al., 1996; Schaufeli & van Dierendonck, 2000; Tuithof et al., 2017) consists of five items: feeling mentally exhausted by work, experiencing a full day’s work as taxing, feeling “burned out” from work, feeling empty at the end of a working day, and feeling tired when facing another workday in the morning. Items were rated on a 7-point frequency scale from 1 (*never*) to 7 (*always/daily*). Mean scores were calculated for respondents with no more than two missing values, with higher scores indicating more severe burnout symptoms. The scale demonstrated good internal consistency in the current study (α = .84).

#### Life Satisfaction Scale

This instrument is methodologically similar to established measures such as the Manchester Short Assessment of Quality of Life (MANSA; Priebe et al., 1999) and/or the Well-being Instrument (WiX; Voormolen et al., 2024). Life satisfaction was measured across seven life domains: work or main activity, family life, relationships with friends, leisure activities, housing, income, and life in general. Each domain was rated on a 5-point scale, with higher scores indicating greater satisfaction across these life domains. Scale scores were computed only for cases with complete data on all items. The scale demonstrated acceptable internal consistency in the current study (α = .78).

#### De Jong Gierveld Loneliness Scale

The De Jong Gierveld Loneliness Scale (De Jong-Gierveld & Kamphuls, 1985) consists of 11 items: six negatively worded items and five positively worded items, with respondents indicating whether statements apply to their recent experiences using a 3-point scale (1 = ”*yes*,” 2 = ”*somewhat*,” 3 = ”no”). For scoring, negative items were considered present if answered with “yes” or “somewhat,” while positive items were considered present if answered with “somewhat” or “no.” The total score ranges from 0 to 11, with higher scores indicating greater loneliness. Respondents were allowed a maximum of one missing item for the total score calculation. The scale demonstrated good internal consistency in the current study (α = .85).

#### The Medical Outcomes Study Short Form-36

The Medical Outcomes Study Short Form-36 (SF-36; Ware & Sherbourne, 1992) assesses health-related quality of life across eight domains: (a) limitations in physical activities because of health problems (Physical Functioning; 10 items; α = .92 in the current study), (b) limitations in social activities because of physical or emotional problems (Social Functioning; 2 items; α = .72 in the current study), (c) limitations in usual role activities because of physical health problems (Role-Physical; 4 items; α = .89 in the current study), (d) limitations in usual role activities because of emotional problems (Role-Emotional; 3 items; α = .86 in the current study), (e) general mental health including psychological distress and well-being (Mental Health; 5 items; α = .81 in the current study), (f) Vitality (4 items; α = .80 in the current study), (g) Bodily Pain (2 items; α = .85 in the current study), and (h) General Health (5 items; α = .76 in the current study). All scales were transformed to scores ranging from 0 to 100, with higher scores indicating better functioning.

### Statistical Analysis

Although the NEMESIS-3 dataset includes sampling weights, we utilized unweighted data throughout our analyses to maintain consistency between our descriptive and psychometric evaluations, as our primary aim was to examine measurement properties rather than establish population norms. All analyses were conducted in R (version 4.4.2), primarily using the lavaan package for structural equation modeling (Rosseel, 2012). To examine the underlying factor structure, we conducted CFA in the total sample using weighted least squares means and variance (WLSMV) adjusted estimation with theta parameterization, appropriate for ordinal data (Brauer et al., 2023). Given previous studies suggesting a one-factor solution (Moeller et al., 2023, 2024; Šaltytė Benth et al., 2023; Williams et al., 2015), a strict unidimensional model was tested with all items loading on a single underlying factor without allowing residual correlations between the items. Model fit was evaluated using the scaled robust versions of the comparative fit index (CFI), root mean square error of approximation (RMSEA), and standardized root mean square residual (SRMR). Following conventional guidelines, we considered CFI > 0.95, RMSEA < 0.06, and SRMR < 0.05 as conservative cutoff criteria for good fit, while CFI > 0.90, RMSEA < 0.10, and SRMR < 0.10 were considered as liberal cutoff criteria for acceptable fit (Van De Schoot et al., 2012). Individual standardized factor loadings >0.50 were considered acceptable (Hair et al., 2019).

Prior to conducting measurement invariance analyses, we examined potential demographic differences between participants with and without CMDs that could influence our findings. For continuous variables (age), independent samples *t*-tests were conducted with Cohen’s *d* calculated as a measure of effect size using pooled standard deviation (Lakens, 2013). For categorical variables (gender and education level), Pearson’s chi-square tests of independence were employed. These univariate comparisons were performed separately for both lifetime CMD status and current (past 12-month) CMD status to identify any systematic demographic differences that should be considered when interpreting measurement invariance results.

To assess measurement invariance across CMD status, we conducted multigroup CFAs, successively testing increasingly strict levels of configural (same factor structure), metric (equal factor loadings), and scalar (equal factor loadings and equal thresholds) invariance based on the one-factor model. As WLSMV estimation does not directly model residual variances, strict invariance was not tested (equal residuals). Model fit at the metric and scalar levels was compared to the previous level. Because (scaled) chi-square difference tests are overly sensitive to sample size, we relied on changes in scaled CFI, RMSEA, and SRMR indices to test invariance at each level. Following Chen (2007), for sample sizes >300, a change of ≥−0.010 in CFI supplemented by a change of ≥ 0.015 in RMSEA or ≥ 0.030 in SRMR would indicate noninvariance at the metric level, while for scalar invariance, a change of ≥−0.010 in CFI supplemented by a change of ≥0.015 in RMSEA or ≥0.010 in SRMR would indicate noninvariance.

Internal consistency reliability was evaluated using McDonald’s omega (ω) with 95% confidence intervals calculated using percentile bootstrapping with 1,000 resamples. McDonald’s omega was chosen over Cronbach’s alpha as it provides unbiased reliability estimates even when factor loadings vary, a condition under which alpha tends to underestimate population reliability (Flora, 2020; Zinbarg et al., 2005). Coefficients <.70 were considered unacceptable, coefficients between .70 and .79 acceptable, between .80 and .89 good, and > .90 excellent (Cicchetti, 1994). Reliability coefficients were calculated separately for the total sample and subgroups of participants with and without lifetime or current CMDs to examine whether the Brief INSPIRE-O showed comparable internal consistency across these groups.

External construct validity was assessed through zero-order Pearson correlations with established measures of mental health and well-being previously described in the Methods section. Correlations were interpreted as small (*r* < .30), medium (*r* = .30–.50), or large (*r* > .50; Cohen, 1988).

## Results

### Sample and Descriptive Statistics

From the initial sample of 5,451 participants, 5,443 provided complete data on all five Brief INSPIRE-O items. The final sample showed an equal sex distribution (49.9% female) with a mean age of 47.7 years (*SD* = 16.5, range 18–77). Based on unweighted data, 47.2% of participants met criteria for a lifetime CMD as assessed with the CIDI 3.0, while 24.8% met criteria for a past 12-month disorder. It should be noted that these percentages differ from previously reported weighted prevalence rates in the NEMESIS-3 study, partly due to the exclusion of participants who did not consent to data sharing with third parties.

Demographic characteristics showed some differences between those participants with and those without current or lifetime CMDs (see Table 1). Participants with a current CMD (*t*(2,285) = −15.7, *p* < .001, *d* = 0.49) or lifetime CMD (*t*(5393) =−12.24, *p* < .001, *d* = −0.33) were on average significantly younger than those with no current or lifetime CMD, respectively. Gender distribution also differed significantly between groups, with a higher proportion of females among participants with a current CMD (χ^2^(1) = 13.07, *p* < .001) or lifetime CMD (χ^2^(1) = 27.78, *p* < .001) compared to those without. Education levels showed no significant differences between groups for either current CMDs (χ^2^(2) = 5.27, *p* = .072) or lifetime CMDs (χ^2^(2) = 4.41, *p* = .110).

**Table 1:** Sample Descriptive Statistics.

Variables	Total sample	No current CMDs	Current CMDs	No lifetime CMDs	Lifetime CMDs
Age, *M* (*SD*)	47.7 (16.5)	49.7 (16.1)	41.7 (16.2)	50.2 (16.3)	44.8 (16.2)
Sex, *n* (%)					
Female	2,721 (49.9%)	1989 (48.5%)	732 (54.2%)	1339 (46.5%)	1382 (53.7%)
Male	2,730 (50.1%)	2112 (51.5%)	618 (45.8%)	1539 (53.5%)	1191 (46.3%)
Education, *n* (%)					
Level 1	1,200 (22.0%)	883 (21.5%)	317 (23.5%)	630 (21.9%)	570 (22.2%)
Level 2	2,027 (37.2%)	1510 (36.8%)	517 (38.3%)	1038 (36.1%)	989 (38.4%)
Level 3	2,224 (40.8%)	1708 (41.6%)	516 (38.2%)	1210 (42.0%)	1014 (39.4%)
Brief INSPIRE-O Total Score, *M* (*SD*)	74.79 (14.10)	76.72 (12.47)	68.93 (16.89)	77.34 (12.40)	71.94 (15.30)

*Note*. M = mean; SD = standard deviation; CMDs = common mental disorders; Level 1 = Primary education, lower secondary; Level 2 = Higher secondary; Level 3 = Higher vocational, university.

Total Brief INSPIRE-O scores were reasonably normally distributed but negatively skewed, with most respondents scoring relatively high in the 0 to 100 range. Mean Brief INSPIRE-O total scores differed significantly between diagnostic groups. Participants without lifetime CMDs scored higher (*M* = 77.34, *SD* = 12.40) than those with lifetime CMDs (*M* = 71.94, *SD* = 15.30; *t*(4,950.7) = 14.21, *p* < .001, *d* = 0.39). Similarly, those without current CMDs showed higher scores (*M* = 76.72, *SD* = 12.47) compared to those with current CMDs (*M* = 68.93, *SD* = 16.89; *t*(1,857.2) = 15.61, *p* < .001, *d* = 0.57). Descriptive statistics for the item-level response frequencies of the Brief INSPIRE-O can be found in the Supplemental Table.

### Factor Structure

CFA in the total sample supported a one-factor structure with good model fit according to the CFI and SRMR, but unacceptable fit according to the RMSEA (scaled χ^2^(df) = 460.01(5), CFI = 0.968, SRMR = 0.045, RMSEA = 0.129 (90% CI [0.119, 0.139]), robust RMSEA = 0.120 [0.109, 0.131]). Examination of modification indices (MI) revealed that the largest improvement (*MI* = 220.31) would result from adding an error correlation between item 1 (“I feel supported by other people”) and item 2 (“I have hopes and dreams for the future”). A modified model with this correlation demonstrated excellent fit across all indices (scaled χ^2^(df) = 26.34(4), CFI = 0.998, SRMR = 0.010, RMSEA = 0.032 [0.021, 0.044], robust RMSEA = 0.032 [0.020, 0.045]). However, given the briefness of the 5-item scale and potential for overfitting without clear theoretical justification for item-specific error correlations, we retained the original one-factor model for all subsequent invariance analyses to maintain model parsimony and generalizability. Standardized factor loadings in the unmodified strict unidimensional model ranged from 0.566 to 0.810 (see Table 2), indicating adequate measurement quality of all five individual items despite the elevated RMSEA.

**Table 2:** Standardized Factor Loadings for the One-Factor Model of the Brief INSPIRE-O in the Total Sample (*N* = 5,443).

Item	Loading	*R*¹
I feel supported by other people	0.566	0.320
I have hopes and dreams for the future	0.594	0.353
I feel good about myself	0.810	0.656
I do things that mean something to me	0.749	0.561
I feel in control of my life	0.712	0.507

*Note.* All factor loadings are significant at *p* < .001.

R^2^ represents the proportion of variance in each item explained by the latent factor.

### Measurement Invariance

Having verified the unidimensionality of the Brief INSPIRE-O, we estimated successively stricter measurement invariance models between groups with and without lifetime CMDs. Adding the constraints of equal factor loadings (metric invariance) maintained good fit. The change in scaled fit indices was minimal (ΔCFI = 0.009, ΔRMSEA = −0.036, ΔSRMR = 0.000) with a non-significant scaled χ^2^ difference test (Δχ^2^ = −121.37), supporting metric invariance. This indicates that the items relate to the latent construct in the same way across groups. Further constraining item thresholds to be equal (scalar invariance) showed largely supportive results. While the ΔCFI (−0.011) marginally exceeded the recommended cutoff of −0.010, both ΔRMSEA (−0.013) and ΔSRMR (0.001) remained well within acceptable ranges. Following Chen’s (2007) recommendation to evaluate measurement invariance holistically using multiple indices rather than relying on strict cutoffs of any single index, and considering that the ΔCFI violation was minimal, we concluded that scalar invariance was supported. Tests of measurement invariance across people with or without current CMDs showed similar results, supporting both metric and scalar invariance (see Table 3).

**Table 3:** Measurement Invariance Tests of the Brief INSPIRE-O Between Groups With and Without Lifetime And Current CMDs.

Model	CFI	RMSEA	Robust RMSEA	SRMR	χ¹	*df*	ΔCFI	ΔRMSEA	ΔSRMR	Δχ¹
Lifetime CMDs										
Configural	0.967	0.129	0.122	0.045	461.45	10	—	—	—	—
Metric	0.976	0.093	0.103	0.045	340.08	14	0.009	−0.036	0.000	−121.37
Scalar	0.965	0.080	—	0.046	513.99	28	−0.011	−0.013	0.001	173.91*
Current CMDs										
Configural	0.966	0.129	0.121	0.046	459.57	10	—	—	—	—
Metric	0.975	0.094	0.103	0.047	348.13	14	0.009	−0.035	0.001	−111.44
Scalar	0.967	0.076	—	0.047	472.69	28	−0.008	−0.018	0.000	124.55*

*Note.* CMDs = common mental disorders; CFI = comparative fit index; RMSEA = root mean square error of approximation; SRMR = standardized root mean square residual.

* *p* < .05.

These results indicate that the Brief INSPIRE-O demonstrates strong measurement invariance between groups with and without CMDs, meaning observed score differences between these groups reflect true differences in the underlying construct.

### Internal Consistency Reliability

The Brief INSPIRE-O demonstrated acceptable reliability in the total sample (ω = .75, 95% CI [0.74, 0.77]). Reliability estimates were comparable between participants without lifetime CMDs (ω = .72, [0.70, 0.74]) and those with lifetime CMDs (ω = .76, [0.74, 0.78]). Similar patterns were observed between those without current CMDs (ω = .71, [0.69, 0.73]) and those with current CMDs (ω = .78, [0.76, 0.80]).

### External Construct Validity

The Brief INSPIRE-O showed medium to large associations with constructs conceptually related to psychological well-being in the total sample (see Table 4). As expected, life satisfaction showed a large positive correlation (*r* = .56), while loneliness demonstrated a near-large negative association (*r* = −.49). Similarly, mental health-related domains of the SF-36 exhibited large correlations (mental health: *r* = .54; vitality: *r* = .50), while social functioning (*r* = .36) and role-emotional functioning (*r* = .31) demonstrated an expected medium correlation. As expected, physical health-related measures showed the weakest correlations (physical functioning: *r* = .25; role-physical: *r* = .24; bodily pain: *r* = .23). Similar small to medium correlations were observed with work loss and burnout, supporting the discriminant validity of the scale.

**Table 4:** Correlations Between Brief INSPIRE-O and Other Variables Across Different Groups.

Variable	Total sample	No lifetime disorder	Lifetime disorder	No current disorder	Current disorder
	(*N* = 5,451)	(*n* = 2,878)	(*n* = 2,572)	(*n* = 4,100)	(*n* = 1,350)
Work loss	−.25	−.13	−-.28	−.13	−.33
Burnout	−.32	−.25	−.32	−.25	−.34
Loneliness	−.49	−.37	−.53	−.37	−.60
Life satisfaction	.56	.44	.61	.45	.67
Physical functioning (SF-36)	.25	.23	.24	.20	.30
Social functioning (SF-36)	.36	.23	.41	.22	.48
Role-physical (SF-36)	.24	.18	.24	.16	.31
Role-emotional (SF-36)	.31	.10	.37	.11	.41
Mental health (SF-36)	.54	.41	.59	.41	.64
Vitality (SF-36)	.50	.40	.52	.40	.56
Bodily pain (SF-36)	.23	.19	.23	.17	.27
General health (SF-36)	.45	.40	.45	.37	.51

*Note.* All correlations are significant at *p* < .001.

SF-36 = Medical Outcomes Study Short Form-36.

This pattern of correlations was consistently stronger in participants with mental disorders, particularly those with current disorders. For example, correlations with life satisfaction (*r* = .67 vs. *r* = .45), mental health (*r* = .64 vs. *r* = .41), and loneliness (*r* = −.60 vs. *r* = −.37) were notably stronger in the current disorder group.

## Discussion

The current study aimed to validate the Brief INSPIRE-O further and to assess measurement invariance across people with and without a history of CMDs in a large sample of the Dutch general population (Ten Have, Tuithof, Van Dorsselaer, Schouten, & De Graaf, 2023). Overall, the items of the Brief INSPIRE-O measured a single underlying construct with good factor loadings for all items in the total sample. The unidimensional model demonstrated scalar invariance between people with and without a lifetime or current mental disorder, indicating that the scale measures the same underlying construct in these groups. Finally, internal consistency reliability was acceptable across both people with and without mental disorders, and total scores largely demonstrated the hypothesized pattern of correlations with measures of health and well-being.

First, a CFA was conducted in the total sample to examine the underlying single-factor structure. Our findings supported a unidimensional structure of the Brief INSPIRE-O, suggesting that the five CHIME domains function as related aspects of a single underlying construct of personal recovery. While most fit indices supported this structure, the RMSEA indicated some misfit, due to our use of a strict unidimensional model without residual correlations (Goretzko et al., 2024). Despite this technical limitation, the strong factor loadings and overall model fit provide confidence in treating the Brief INSPIRE-O as a unidimensional measure, consistent with previous validations of the scale (Moeller et al., 2023, 2024). This unidimensional structure has practical advantages, offering clinicians and researchers a straightforward way to assess personal recovery while still capturing its multifaceted nature through the five CHIME domains.

Second, measurement invariance across people with (current or lifetime) and without CMD was assessed using multigroup CFA. Our analyses demonstrated that the Brief INSPIRE-O measures personal recovery equivalently in people with and without CMDs. This measurement invariance is crucial as it indicates that the concept of personal recovery is understood and experienced similarly across these groups, even though their actual levels may differ. In practical terms, this means we can meaningfully compare Brief INSPIRE-O scores between people with and without CMDs, knowing that observed differences reflect true variations in personal recovery rather than differences in how the construct is measured or understood. This finding supports the broader theoretical perspective that personal recovery represents aspects of psychological well-being that are relevant to all individuals, regardless of their mental health status (Kraiss et al., 2019; Van Eck et al., 2023). Such measurement invariance is particularly valuable for researchers studying recovery processes across different populations and for clinicians tracking recovery progress in relation to general population norms. Notably, while our multigroup CFA approach confirms measurement invariance at the total *scale* level, alternative methods such as differential item functioning (DIF) analysis based on item response theory (IRT) can detect noninvariance more precisely at the *item* level. Such methods may uncover subtle item-level biases even when the overall measurement model appears equivalent, and thus might yield different results (Stark et al., 2006). Future studies could employ IRT or multiple indicators multiple causes modeling to examine the presence of both uniform and nonuniform DIF in more detail. In addition, given that participants with and without CMDs differed significantly in gender and age composition in the present study, future research should specifically examine measurement invariance across different demographic subgroups.

Third, associations with work loss, burnout, life satisfaction, loneliness, and health-related quality of life were assessed to further examine the construct validity of the Brief INSPIRE-O. As expected, personal recovery showed particularly strong connections with aspects of psychological and social functioning, such as life satisfaction, mental health, and loneliness. A notable finding was that these relationships were consistently stronger among people with current CMDs compared to those without such disorders. This differential pattern of associations has important implications for understanding recovery processes. It suggests that personal recovery may become more closely intertwined with multiple domains of functioning for individuals currently experiencing mental health difficulties. These findings extend previous validations of the Brief INSPIRE-O (Moeller et al., 2023, 2024) and contribute to the broader literature emphasizing the importance of addressing personal recovery in mental health care (Saavedra et al., 2023; Slade, 2010; Winefield et al., 2012). However, the cross-sectional nature of our study limits causal interpretations of these associations. While personal recovery may facilitate better functional outcomes, it is equally plausible that improved functioning in various life domains contributes to greater experiences of personal recovery.

Overall, the findings of the current study establish the Brief INSPIRE-O as a valid and reliable instrument for measuring personal recovery or psychological well-being across individuals with and without CMDs. Observed score differences reflect genuine variations in positive psychological functioning rather than measurement artifacts. These findings align with theoretical frameworks positing substantial overlap between the CHIME dimensions of personal recovery and established constructs of psychological well-being (Kraiss et al., 2019; Resnick & Rosenheck, 2006; Ryff, 1989; Ryff & Keyes, 1995; Slade, 2010; Van Eck et al., 2023), supporting the conceptualization of personal recovery as a universal psychological construct rather than one exclusive to clinical populations.

Our findings contribute to the evidence base of personal recovery measures. The comprehensive systematic review by Felix et al. (2024) examined 25 patient-reported outcome measures (PROMs) of personal recovery, although this review notably did not include the Brief INSPIRE-O. The review identified significant gaps in the psychometric evidence base for existing PROMs, particularly regarding measurement invariance. Our validation of the Brief INSPIRE-O thus contributes new psychometric evidence to the available personal recovery self-report measures. While Leamy et al. (2023) found that the original INSPIRE demonstrated the most extensive psychometric testing among recovery-oriented measures, our findings provide further psychometric evidence for the Brief INSPIRE-O as an adaptation for measuring self-perceived levels of personal recovery. Importantly, our demonstration of measurement invariance between individuals with and without CMDs supports the use of the Brief INSPIRE-O not only as a recovery measure but also as a general psychological well-being assessment tool. As the Brief INSPIRE-O consists of only 5 items, it could address a need for a feasible, yet psychometrically sound, measure that can be used in both routine and research settings. This is also illustrated by recent studies that already included the Brief INSPIRE-O in their battery of outcome measures (Labourot et al., 2024; Jakobsen et al., 2025).

### Strengths and Limitations

Our study is the first to thoroughly assess the psychometric properties of Brief INSPIRE-O in a large Dutch general population sample. This methodological strength enabled robust comparisons between people with and without a history of mental disorders. In addition, all participants completed a fully structured face-to-face diagnostic interview using the CIDI 3.0. While this instrument has demonstrated good validity for assessing DSM-IV disorders, it should be noted that the validity and reliability of the current slightly modified CIDI 3.0 for DSM-5 diagnoses have not been formally investigated (Ten Have, Tuithof, Van Dorsselaer, Schouten, Luik, et al., 2023). One important limitation of the current study is that people with severe mental disorders were underrepresented in the current sample, as individuals who were institutionalized (in hostels, hospices, or prisons) were excluded from the study (Ten Have, Tuithof, Van Dorsselaer, Schouten, & De Graaf, 2023). This limitation becomes particularly evident when comparing our findings with those of Swildens et al. (2025), who recently validated the Dutch Brief INSPIRE-O specifically in individuals with severe mental illness receiving treatment. Their sample reported notably lower personal recovery scores (*M* = 58.14, *SD* = 18.59) than our participants with current CMDs (*M* = 68.93, *SD* = 16.89), highlighting potential differences in how personal recovery is experienced across the severity spectrum of mental health conditions. Therefore, our findings regarding measurement invariance cannot be confidently generalized to populations with severe mental illness in institutional settings. A second limitation is that our analysis did not examine test–retest reliability and longitudinal measurement invariance of the Brief INSPIRE-O, despite the availability of follow-up data in the NEMESIS-3 cohort. We omitted these analyses because they extend beyond our primary research aims, although exploring them in future studies could further validate the instrument.

## Conclusion

The Brief INSPIRE-O appears to be a reliable and valid instrument to measure personal recovery and individual psychological well-being in people with and without a current or a history of CMDs.

## Supplemental Material

sj-docx-1-asm-10.1177_10731911251367122 – Supplemental material for Personal Recovery in the General Population: Comparison of Psychometric Properties of the Brief INSPIRE-O in Those With and Without Common Mental Disorders Supplemental material, sj-docx-1-asm-10.1177_10731911251367122 for Personal Recovery in the General Population: Comparison of Psychometric Properties of the Brief INSPIRE-O in Those With and Without Common Mental Disorders by Xingjian Ruan, Femke Vergeer-Hagoort, Margreet ten Have, Annemarie I. Luik, Marlous Tuithof, Ernst Bohlmeijer and Peter M. ten Klooster in Assessment
