# Supplementary material for: Personal Recovery in the General Population: Comparison of Psychometric Properties of the Brief INSPIRE-O in Those With and Without Common Mental Disorders
Source: Assessment. 2025 Sep 16;33(6):1000–11. doi: 10.1177/10731911251367122 (PMC13379615; doi:10.1177/10731911251367122)
Supplement: sj-docx-1-asm-10.1177_10731911251367122 – Supplemental material for Personal Recovery in the General Population: Comparison of Psychometric Properties of the Brief INSPIRE-O in Those With and Without Common Mental Disorders [file sj-docx-1-asm-10.1177_10731911251367122.docx]

Supplementary Table. Item-level response frequencies for the Brief INSPIRE-O

| Brief INSPIRE-O Items | Not at all | Not much | Somewhat | Quite a lot | Very much |
| --- | --- | --- | --- | --- | --- |
| Item 1 "I feel supported by other people" | 61 (1.1%) | 125 (2.3%) | 656 (12.0%) | 2,652 (48.7%) | 1,955 (35.9%) |
| Item 2 "I have hopes and dreams for the future" | 89 (1.6%) | 327 (6.0%) | 1,121 (20.6%) | 2,564 (47.1%) | 1,348 (24.7%) |
| Item 3 "I feel good about myself" | 37 (0.7%) | 160 (2.9%) | 973 (17.9%) | 3,181 (58.4%) | 1,095 (20.1%) |
| Item 4 "I do things that mean something to me" | 15 (0.3%) | 117 (2.1%) | 720 (13.2%) | 3,200 (58.7%) | 1,397 (25.6%) |
| Item 5 "I feel in control of my life" | 73 (1.3%) | 194 (3.6%) | 1,003 (18.4%) | 3,052 (56.0%) | 1,128 (20.7%) |
